# Catalytic hydrogenation of acetone to isopropyl alcohol with CAl_3_MgH_2_^¯^ containing planar tetracoordinate carbon

**DOI:** 10.3389/fchem.2025.1750798

**Published:** 2026-01-13

**Authors:** Abdul Hamid Malhan, Krishnan Thirumoorthy

**Affiliations:** 1 Department of Chemistry, School of Advanced Sciences, Vellore Institute of Technology, Vellore, Tamil Nadu, India; 2 School of Computer Science and Engineering, Vellore Institute of Technology, Vellore, Tamil Nadu, India

**Keywords:** acetone hydrogenation, CAl₃MgH₂⁻, density functional theory, isopropyl alcohol, planar tetracoordinate carbon

## Abstract

The current study, employing density functional theory, reports the hydrogenation of acetone to isopropyl alcohol catalyzed by CAl_3_MgH_2_^¯^, which contains a planar tetracoordinate carbon (ptC). Various computational approaches are employed to analyze acetone hydrogenation using the CAl_3_MgH_2_^¯^ as a potential catalyst. The reaction initiates with the carbonyl insertion into the Mg–H bond of the CAl_3_MgH_2_^¯^, followed by hydrogenation using molecular hydrogen (H_2_). Analysis of natural atomic charges confirms that the H_2_ molecule dissociates heterolytically into a proton−hydride pair, thereby regenerating the CAl_3_MgH_2_^¯^ in the product state. Intrinsic reaction coordinate calculations confirm the true connection between the reactant and product in the reaction pathway. This investigation highlights the potential of the ptC molecule as a catalyst and delineates the way for new opportunities in ptC-based catalysts.

## Introduction

In recent years, the global market has experienced a surplus of acetone, primarily due to increased production from the cumene process used in phenol synthesis and bio-butanol fermentation (Papa, 2000; Weber and Weber, 2010). This increasing production of acetone, coupled with stable demand for its conventional derivatives like bisphenol A and methyl methacrylate, has led to a market surplus. This economic imbalance necessitates the valorization of excess acetone by converting it into more valuable chemicals. The catalytic hydrogenation of acetone to isopropyl alcohol is a promising and economically sustainable route to address this surplus while simultaneously meeting the rising demand for isopropyl alcohol (Mawarnis and Umar, 2019; Yang et al., 2017). The catalytic hydrogenation of acetone to synthesize isopropyl alcohol has been extensively investigated in both gas-phase (Al-Rabiah et al., 2022; Shen et al., 2021) and liquid-phase (Rahman and S-Al-Deyab, 2014). In gas-liquid hydrogenation reactions, the reaction rate can be limited by the mass transfer of the gaseous reactant (H_2_) into the liquid phase. The low solubility of H_2_ in organic solvents poses a significant challenge for liquid-phase hydrogenation, often requiring elevated pressures to achieve a sufficient dissolved hydrogen concentration at the catalyst surface. Conversely, gas-phase catalysis effectively circumvents this fundamental mass-transfer limitation. By vaporizing both reactants and passing them over a catalyst, the system operates in a single phase, ensuring intimate contact among hydrogen, acetone, and the catalyst and thereby eliminating the gas-liquid interfacial barrier. The gas-phase catalytic hydrogenation of acetone to isopropyl alcohol is a strategically vital, environmentally benign chemical transformation that offers economic feasibility and attractiveness. Furthermore, the application of gas-phase catalysis offers substantial process intensification benefits compared to traditional liquid-phase systems, including the mitigation of mass-transfer limitations and the ability to operate at milder, near-ambient pressures. This leads to enhanced selectivity and simplified product separation, positioning it as a key technology for modern and sustainable chemical manufacturing. In 2022, Al-Rabiah et al. reported the gas-phase catalytic hydrogenation of acetone to isopropyl alcohol using a composite ruthenium on activated charcoal and nano-zinc oxide catalyst, achieving high selectivity (Al-Rabiah et al., 2022). The catalytic hydrogenation of carbonyl derivatives using molecular hydrogen is a vital process in modern synthetic organic chemistry and the production of both bulk and fine chemicals (Rylander, 1979). This type of hydrogenation typically involves well-defined noble metal complexes such as those of ruthenium, rhodium, or iridium (Chirila et al., 2024; Dub and Ikariya, 2012; Johnson et al., 2007; Noyori and Ohkuma, 2001). However, limited availability, substantial cost, and the toxicity of precious metals have sparked interest in developing more sustainable hydrogenation methods. As a result, there is a growing focus on identifying earth-abundant, main-group metals that can potentially replace these noble counterparts (Harder, 2012; Hill et al., 2016; Magre et al., 2022; Revunova and Nikonov, 2015; Roy et al., 2021). The development of well-defined main-group metal-based catalysts with comparable activity holds great promise for future industrial applications in chemistry.

In recent years, there has been a significant focus on magnesium-based catalysis, particularly since the pioneering structural characterization of Mg-hydride complexes (Bonyhady et al., 2010). Magnesium-based catalysts have shown promise in various catalytic reactions, including hydrosilylation (Garcia et al., 2019a; Garcia et al., 2019b; Rauch et al., 2017), hydroboration (Arrowsmith et al., 2012; Magre et al., 2019; Mukherjee et al., 2014; Szewczyk et al., 2019), hydroamination (Dunne et al., 2010; Zhang et al., 2012), hydrogenation (Liang et al., 2022), and dehydrocoupling (Liptrot et al., 2014; Ried et al., 2019; Wirtz et al., 2022) marking considerable progress in this field. The magnesium-based catalyst has proven to be significant in asymmetric catalysis (Wang et al., 2024; Yang et al., 2019), with applications in asymmetric thia-Michael addition (Jaszczewska-Adamczak et al., 2024), asymmetric hydroalkylation (Ye et al., 2025), and asymmetric hydroboration (Falconnet et al., 2019). One of the primary challenges in using main-group metal compounds as catalysts is their limited ability to activate H_2_ bonds and their high reactivity, which often leads to unexpected side reactions. Therefore, it is crucial to develop stable main-group metal complexes with enhanced catalytic activity to address these issues. Both experimental and theoretical studies have highlighted stable main-group molecules with planar hypercoordination (Das et al., 2023; Inostroza et al., 2023; Leyva-Parra et al., 2021; Sun et al., 2020; Sun et al., 2022; Wang et al., 2023; Yang et al., 2015; Zhang et al., 2021), offering a potential solution. The study of planar tetracoordinate carbon (ptC) has attracted significant interest since its initial discovery in 1968 by H. J. Monkhorst (Monkhorst, 1968). Experimentally identified species such as CAl_4_^2–^, CAl_4_^¯^, and C_2_Al_5_^¯^ containing ptC have been reported (Li et al., 1999; 2000; Tsuruoka et al., 2018; Zhang et al., 2023). Additionally, C_2_Al_4_^¯^ and C_5_Al_5_^¯^, containing ptC, were initially computationally predicted, subsequently leading to their experimental identification (Naumkin, 2008; Wu et al., 2011; Zhang et al., 2021; Zhang et al., 2023). The global minimum ptC of CAl_4_H^0/–^ containing hydrogen was both experimentally and theoretically reported (Xu et al., 2017). Additionally, planar hypercoordinate systems of CAl_*n*_Be_*m*_H_*x*_^*q*^ (*n* + *m* = 5, *q* = 0, ±1, *x* = *q* + *m* – 1), Al_2_C_4_H_2_, CBe_2_H_5_^¯,^ and Si_2_C_5_H_2_ containing hydrogen have also been reported (Jin et al., 2024; Malhan et al., 2022; Thirumoorthy et al., 2020; Zhao et al., 2018). The application of planar hypercoordinate carbon systems for reversible hydrogen storage properties was reported in 2023 (Sarmah et al., 2023). Notably, it was found that the planar hypercoordinate carbon systems of CSi_2_Li_2_, CBe_5_Li_5_^+^, and CS_3_Li_3_^+^ exhibit high gravimetric densities of 25.6, 39.7, and 13.5 wt% for hydrogen storage. Additionally, the research suggested potential applications of planar hypercoordinate systems in optoelectronics, electronics, and photovoltaics (Yang et al., 2015). In 2025, Zhang et al. (2025) computationally reported the ptC catalysts C_2_B_2_Me_2_ and C_2_B_2_ *t*Bu_2_ in thermodynamically stable Au^I^ complexes, which were examined for their catalytic activity in the allylic acetate rearrangement. It demonstrated that the ptC catalyst (C_2_B_2_Me_2_)Au^I^ significantly outperforms the conventional N-heterocyclic carbene–Au^I^ system in the allylic acetate rearrangement. This superior activity stems from a considerably lower energy barrier, indicating that the reaction is both kinetically and thermodynamically more favorable with the ptC catalyst. This enhanced performance is directly related to the ptC’s unique electronic structure, which establishes a more beneficial electronic environment for the Au center throughout the catalytic cycle. Significantly, the inherent planarity and electron delocalization of the ptC function as active contributors rather than mere passive characteristics, thus driving the observed increase in catalytic activity.

Our research on the earth-abundant main-group elements has revealed that ptC is a global minimum structure in CAl_3_MgH_2_^¯^ in its singlet state (Malhan and Thirumoorthy, 2024). Extensive analysis using various quantum chemical tools has confirmed the stability of this structure. The study identified that the terminal hydrogen (−0.71 |e|) attached to magnesium exhibits a higher hydridic nature compared to the bridging hydrogen (−0.40 |e|). The bridging hydrogen, due to its overlap with two aluminum atoms, requires more energy to break a bond, rendering it less reactive than the readily available terminal hydrogen, which has a higher hydridic character. This discovery of hydridic hydrogen in CAl_3_MgH_2_^¯^ has sparked our interest in exploring its potential as a catalyst, thereby expanding the application of ptCs in hydrogenation reactions. Moreover, a recent theoretical study by our research group (Malhan and Thirumoorthy, 2025) reported that CAl_3_MgH_2_^¯^ can catalyze alkyne hydrogenation, underscoring the need for further investigation into its catalytic properties and mechanistic pathways.

Despite previous and ongoing experimental efforts involving ptC, there is a lack of reliable laboratory techniques for synthesizing stable ptC that can be isolated, investigated, and handled under ambient conditions. However, an in-depth exploration of ptC structures could facilitate the development of novel materials with various chemical and physical properties, paving the way for new research areas.

**FIGURE 1:**
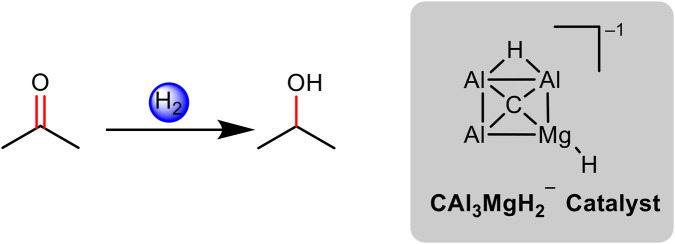
Acetone hydrogenation using CAl_3_MgH_2_^¯^ catalyst.

Given the extensive research and established knowledge in ptC chemistry, viable synthetic methodologies for ptC are anticipated to materialize in the future. Consequently, the importance of the CAl_3_MgH_2_^¯^, which has demonstrated stability, is significantly enhanced, particularly for its potential as a catalyst in hydrogenation reactions. We have addressed the stability of CAl_3_MgH_2_^¯^ in the previous work (Malhan and Thirumoorthy, 2024), the current study reports acetone hydrogenation using CAl_3_MgH_2_^¯^ at room temperature (298.15 K) and 1 atm pressure (Figure 1), supported by quantum chemical calculations.

## Computational methodology

Density Functional Theory (DFT) was utilized to study the hydrogenation of acetone. The geometry optimization was carried out at the ωB97XD/def2-TZVPP (Chai and Head-Gordon, 2008; Weigend and Ahlrichs, 2005; Zheng et al., 2011) level, and harmonic vibrational frequency analysis was performed at the same level to confirm whether the structures represent minima or transition states on the potential energy surface. The ωB97XD functional incorporates a long-range correction term to enhance the description of van der Waals interactions, particularly for noncovalent interactions, dispersion forces, and molecular properties. The def2-TZVPP basis set comprises a triple-zeta valence with polarization functions, providing a detailed electron description and effectively capturing non-covalent interactions. Additionally, all optimized geometries at the ωB97XD/def2-TZVPP level underwent optimization, and harmonic frequency analysis at meta-hybrid M06/def2-TZVPP level (Weigend and Ahlrichs, 2005; Zhao and Truhlar, 2008; Zheng et al., 2011) to ensure that the energy trends were consistent with the results obtained using the former method. To further validate the energetics obtained from the ωB97XD/def2-TZVPP level, single-point energy calculations were subsequently conducted using the DLPNO-CCSD(T)/def2-TZVPP (Riplinger et al., 2013; Riplinger and Neese, 2013) method on the optimized geometries of the ωB97XD/def2-TZVPP level, which was performed using the ORCA program (Neese, 2012). The transition state structures were identified using the Berny algorithm (Schlegel, 1982) within the Gaussian 16 package, which is designed to optimize for a transition state rather than a local minimum. This is achieved through a quasi-Newton method that approximates the Hessian (the second derivative matrix). This method enables the identification of a stationary point on the potential energy surface, specifically the first-order saddle point (transition state). This critical point is characterized by a Hessian matrix possessing exactly one negative eigenvalue, which corresponds to the reaction coordinate. The optimization protocol is rigorously managed to maintain this single negative eigenvalue throughout the search. Essentially, the algorithm proceeds uphill to locate the transition state, which corresponds to a maximum in one direction and a minimum in all others. Various quantum chemical computational tools were employed to comprehensively characterize the chemical bonding properties along the entire reaction pathway. Intrinsic reaction coordinate (IRC) calculations (Gonzalez and Schlegel, 1989) were performed to determine the minimum-energy reaction pathway connecting the dynamics of a reaction. Furthermore, natural population analysis (Reed et al., 1985) within the natural bond orbital framework (Nobel et al., 2017) was performed using the Gaussian 16 program (Frisch et al., 2016) to obtain natural atomic charges. Moreover, non-covalent interaction (NCI) analysis was performed using Multiwfn 3.8 program (Lu and Chen, 2012). The analyses as mentioned above were carried out at the ωB97XD/def2-TZVPP level, and all the calculations, including optimization, frequency, and IRC, were performed with the Gaussian 16 package (Frisch et al., 2016).

## Results and discussion

The stability of CAl_3_MgH_2_^¯^ has been confirmed through computational studies in the previous study, which emphasized the delocalization of electron density and the presence of double aromaticity (Malhan and Thirumoorthy, 2024). A similar isoelectronic structure, CAl_4_H^¯^ (Xu et al., 2017), has been experimentally identified, indicating that substituting one aluminum atom with an isoelectronic Mg–H unit may facilitate the identification of CAl_3_MgH_2_^¯^.

**FIGURE 2:**
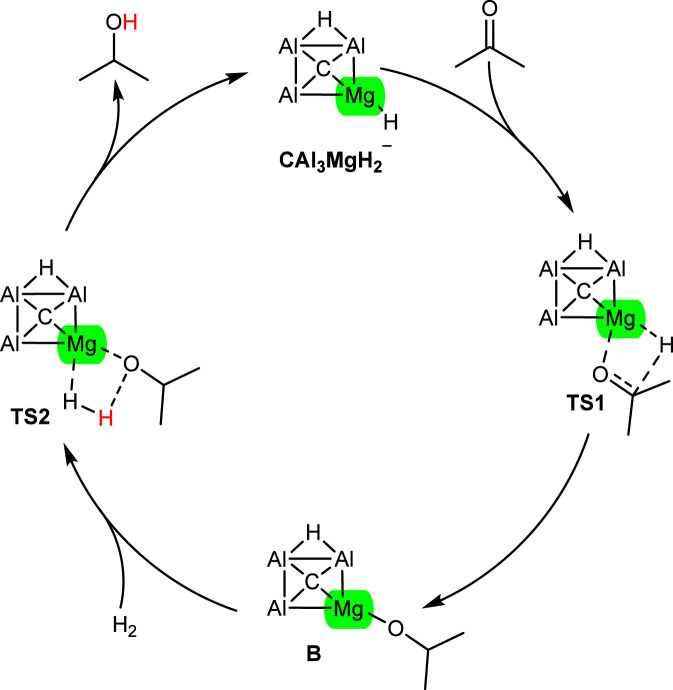
Proposed mechanism for acetone hydrogenation using the CAl_3_MgH_2_^¯^.

The proposed mechanism of acetone hydrogenation using the CAl_3_MgH_2_^¯^ is illustrated in Figure 2. The reaction begins with the coordination of the carbonyl (C═O) to the Mg–H bond. This interaction facilitates the transfer of a hydride ion (H^¯^) from the Mg–H bond to the carbonyl carbon of acetone, proceeding through the transition state TS1. This step results in the formation of an Mg–O bond and produces intermediate B. Subsequently, the introduction of H_2_ leads to the formation of another transition state, TS2. In this step, the H_2_ molecule undergoes cleavage, with one hydrogen atom added to the magnesium atom of the catalyst and another to the oxygen atom. This generates the desired product, isopropyl alcohol, while simultaneously regenerating the CAl_3_MgH_2_^¯^. The present work provides a detailed investigation into the hydrogenation of acetone facilitated by the CAl_3_MgH_2_^¯^, providing valuable insights into the mechanisms involved and the overall efficiency of the catalytic process.

**FIGURE 3:**
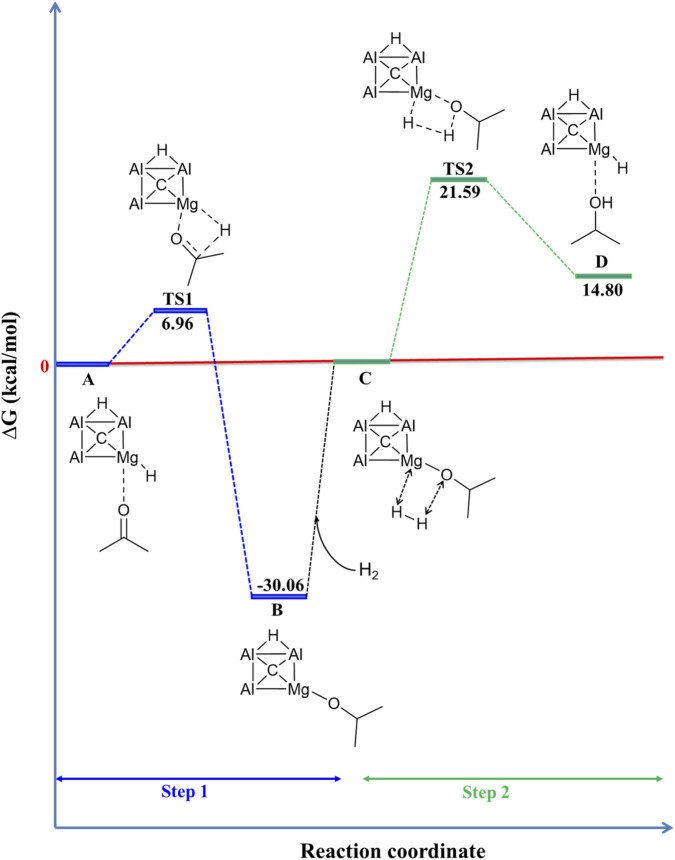
Gibbs free energy profile in kcal/mol for hydrogenation of acetone using CAl_3_MgH_2_^¯^ at the ωB97XD/def2-TZVPP level. The reaction involves two distinct transition states, each with an activation barrier of 6.96 kcal/mol and 21.59 kcal/mol, respectively.

The Gibbs free energy profile depicted in Figure 3 illustrates the hydrogenation of acetone using the CAl_3_MgH_2_^¯^ under standard computational conditions (298.15 K and 1 atm pressure), calculated at the ωB97XD/def2-TZVPP level of theory. Additionally, their zero-point corrected energy profile is displayed in Supplementary Figure S1. Their ZPVE corrections, and the number of imaginary frequencies of all optimized stationary points involved in the hydrogenation of acetone using CAl_3_MgH_2_^¯^ at the ωB97XD/def2-TZVPP and M06/def2-TZVPP levels are listed in Supplementary Table S1 and Supplementary Table S2, respectively. Both density functional methods, transition states TS1 and TS2, each exhibit one negative imaginary frequency, confirming their identity as first-order saddle points on the potential energy surface. Conversely, structures A, B, C, and D possess zero imaginary frequencies, indicating they correspond to minima along the reaction coordinate. The reaction is initiated by the coordination of the carbonyl (C═O) to the Mg–H bond of the CAl_3_MgH_2_^¯^, in reactant state A. Subsequently, carbonyl (C═O) is inserted into the Mg–H bond with an energy barrier of 6.96 kcal/mol, forming a stable intermediate B with −30.06 kcal/mol. Following this, the hydrogenation of the Mg–O bond with molecular hydrogen (H_2_) leads to heterolytic H_2_ cleavage via a four-center transition state (TS2) with an energy barrier of 21.59 kcal/mol. This step, which regenerates the CAl_3_MgH_2_^¯^ and forms the desired product, isopropyl alcohol, in product state D, is the slowest in the reaction. The energy values obtained at the ωB97XD/def2-TZVPP level exhibit a notable similarity with the energy trends observed at the M06/def2-TZVPP level. A comparison of their respective Gibbs free energy and Zero-point corrected energy values is given in Supplementary Table S3. Specifically, the energy values for the transition states (TS1 and TS2) at the M06/def2-TZVPP level indicate a lower activation energy barrier compared to those at the ωB97XD/def2-TZVPP level. However, the energy values across both methodologies remain highly comparable. This alignment highlights the methodological consistency between these two distinct functionals in assessing the reaction energy profile. Such marked consistency across these two different functionals not only enhances the robustness of the computational predictions but also reinforces the credibility of the methodologies employed in this study. Furthermore, the comparison of ΔE values calculated at ωB97XD/def2-TZVPP and CCSD(T)/def2-TZVPP//ωB97XD/def2-TZVPP levels is given in Table 1. The results confirm that the reaction energy trends remain consistent and comparable between the two methods. This further provides strong evidence that the ωB97XD/def2-TZVPP method describes the reaction energetics with near accuracy.

**TABLE 1:** Comparison of ΔE calculated at the ωB97XD/def2-TZVPP and CCSD(T)/def2-TZVPP//ωB97XD/def2-TZVPP levels for hydrogenation of acetone using CAl_3_MgH_2_^¯^.

Structure labels	Energy (kcal/mol)	
	ωB97XD/def2-TZVPP	DLPNO-CCSD(T)/def2-TZVPP//ωB97XD/def2-TZVPP
	ΔE	ΔE
A	0.00	0.00
TS1	6.58	5.89
B	−32.94	−34.76
C	0.00	0.00
TS2	17.95	17.92
D	8.47	8.15

**FIGURE 4:**
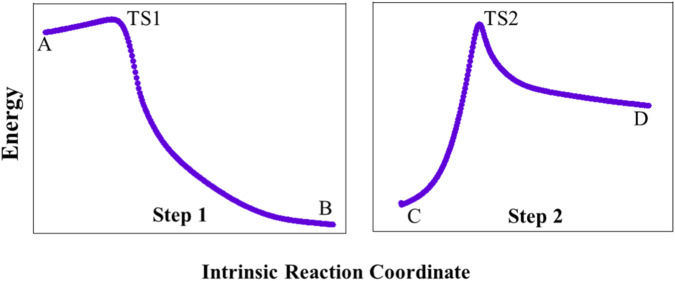
Intrinsic reaction coordinate pathway for hydrogenation of acetone using CAl_3_MgH_2_^¯^. Both the transition states are truly connected to their adjacent local minima. Intrinsic reaction coordinate calculations are performed at the ωB97XD/def2-TZVPP level (Intrinsic reaction coordinate video is provided in the Supplementary Material).

The identification of transition states was achieved through the Berny algorithm implemented in the Gaussian 16 package (Frisch et al., 2016), with a specific focus on the hydrogenation process of acetone catalyzed by CAl_3_MgH_2_^¯^. To gain deeper insights into reaction dynamics, IRC calculations were employed, enabling a comprehensive exploration of the minimum-energy pathways associated with the chemical reaction. These calculations are crucial for elucidating the reaction pathway, providing a comprehensive overview of how reactants are systematically converted into products. The IRC calculations were performed using the transition states TS1 and TS2 identified by the Berny algorithm, and their IRC pathways are given in Figure 4. This analysis confirmed that these transition states are strongly interconnected with both the reactants and the products, thus reinforcing the overall integrity of the proposed reaction pathway. The findings demonstrate that the calculated pathway is energetically feasible, ensuring a reliable depiction of the hydrogenation process.

**FIGURE 5:**
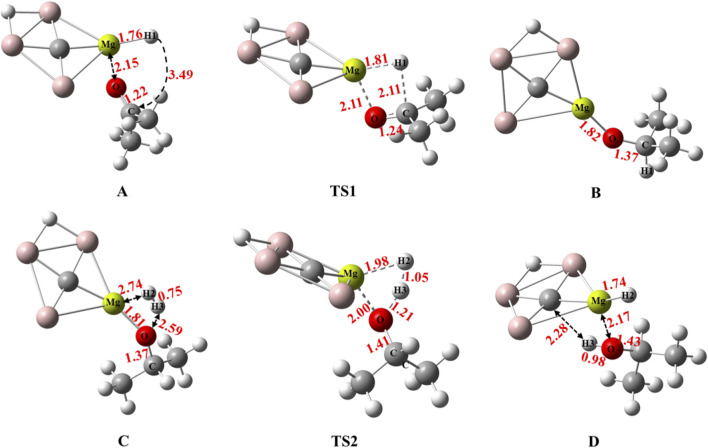
Calculated bond lengths in Å for optimized structures of the stationary points along the reaction pathway for hydrogenation of acetone using CAl_3_MgH_2_^¯^ at the ωB97XD/def2-TZVPP level. The elongation of the Mg–H1 bond in TS1 confirms the transfer of H1. In TS2, the elongation of H_2_ confirms its cleavage.

The optimized geometries on the potential energy surface, including bond lengths, for the hydrogenation of acetone using the CAl_3_MgH_2_^¯^, are given in Figure 5 along with their corresponding natural atomic charges from natural population analysis as provided in Table 2 at the ωB97XD/def2-TZVPP level. Additionally, the optimized geometries are displayed in Supplementary Figure S2 at the M06/def2-TZVPP level. In reactant state A, the hydrogen from the Mg–H1 bond has a natural atomic charge of −0.74 |e|, which indicates its hydridic nature, and the Mg–H1 bond distance is 1.76 Å. As the reaction proceeds, in TS1 the Mg–H1 and C═O bond elongate to 1.81 Å and 1.24 Å, respectively. This elongation facilitates the H1 hydride shift to the carbonyl carbon and the formation of the Mg–O bond. As a result, intermediate B is formed, where the Mg–O bond measures 1.82 Å, and the carbonyl (C═O) reduces into a single C–O bond with a bond length of 1.37 Å. The natural atomic charges on oxygen (−1.10 |e|) and magnesium (1.75 |e|) indicate that they now act as Lewis base and Lewis acid, respectively. This Lewis acid−base pair dissociates H_2_ molecules heterolytically into a proton−hydride pair, thereby favoring the hydrogenation process (Aireddy and Ding, 2022). The natural atomic charges of H2 (−0.45 |e|) and H3 (0.29 |e|) in TS2, in conjunction with the elongation of the H2–H3 bond from 0.75 Å to 1.05 Å, confirm the heterolytic cleavage of H_2_, which leads to the formation of Mg−H2^δ−^ and O−H3^δ+^ pairs. This process results in regenerating the CAl_3_MgH_2_^¯^, and the formation of the desired product, isopropyl alcohol in D. To verify the complete regeneration of the CAl_3_MgH_2_^¯^, the bonding parameters of the CAl_3_MgH_2_^¯^ within the product complex D are compared with those of the isolated CAl_3_MgH_2_^¯^, as shown in Supplementary Figure S3. The results indicate that all bond lengths in the CAl_3_MgH_2_^¯^ of product complex D are identical to those of the isolated CAl_3_MgH_2_^¯^, with no discernible changes. This consistent bond-length measurement provides compelling evidence that the CAl_3_MgH_2_^¯^ has been fully regenerated, thereby preserving its structural integrity. Additionally, a comparison of bond lengths for all optimized structures along the reaction pathway for the hydrogenation of acetone using the CAl_3_MgH_2_^¯^, calculated at the ωB97XD/def2-TZVPP and M06/def2-TZVPP levels, is provided in Supplementary Table S4. Remarkably, the bond lengths calculated from both levels of theory show a high degree of similarity. This consistency in the results reinforces the reliability of the findings across the two theoretical frameworks employed in this study.

**TABLE 2:** Natural atomic charges (|e|) on atoms involved in the reaction pathway of hydrogenation of acetone using CAl_3_MgH_2_^−^ calculated at the ωB97XD/def2-TZVPP and M06/def2-TZVPP levels. (Atom labels are followed as given in Figure 5).

Structure labels	Methods	Mg	O	C	H1	H2	H3
A	ωB97XD/def2-TZVPP	1.58	−0.63	0.64	−0.74	—	—
	M06/def2-TZVPP	1.58	−0.65	0.64	−0.74	—	—
TS1	ωB97XD/def2-TZVPP	1.64	−0.75	0.62	−0.65	—	—
	M06/def2-TZVPP	1.63	−0.76	0.64	−0.64	—	—
B	ωB97XD/def2-TZVPP	1.75	−1.10	0.13	0.12	—	—
	M06/def2-TZVPP	1.74	−1.10	0.14	0.11	—	—
TS2	ωB97XD/def2-TZVPP	1.67	−0.94	0.09	0.16	−0.45	0.29
	M06/def2-TZVPP	1.65	−0.93	0.10	0.15	−0.48	0.31
D	ωB97XD/def2-TZVPP	1.57	−0.83	0.08	0.18	−0.71	0.54
	M06/def2-TZVPP	1.56	−0.84	0.08	0.18	−0.70	0.54

The functional significance of the ptC resides in its unique electronic configuration, which confers absolute planarity even within the transition states. As Figure 5 illustrates, the structural integrity of the ptC core is maintained throughout the reaction. This structural integrity is not merely geometric but stems directly from the extensive electron delocalization in the planar CAl_3_MgH_2_^¯^ core. Critically, this delocalization prevents distortion of the CAl_3_MgH_2_^¯^ core throughout the reaction. Furthermore, the central ptC facilitates catalytic activity by charge stabilization, effectively compensating for charge perturbations at the magnesium active site. This is supported by the natural atomic charges (Table 2), which exhibit minimal variation in the magnesium natural charges throughout the reaction, confirming that the ptC core efficiently prevents destabilizing charge accumulation and effectively stabilizes the transition state.

**FIGURE 6:**
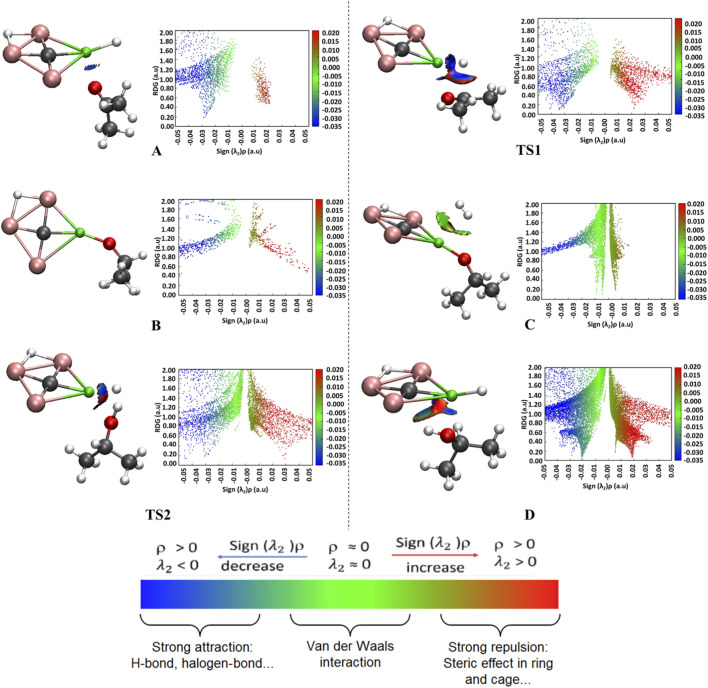
NCI, 3D isosurfaces (on left) and 2D-RDG graphs (on right) for the specific interactions associated with bond breaking and formation involved in the reaction pathway of hydrogenation of acetone using CAl_3_MgH_2_^¯^ at ωB97XD/def2-TZVPP level. The reaction initiates with van der Waals interactions (green isosurface) that bring the reactants together in the initial complex. As the system approaches the transition state, these evolve into strong, electrostatic interactions (blue isosurface), which are critical for product formation. Isosurfaces are colored as: strong attraction (blue), van der Waals interaction (green), and repulsive interaction (red). The complete 3D isosurfaces and their corresponding RDG graphs are given in Supplementary Figure S4.

The NCI analysis was performed using converged Self-Consistent Field (SCF) calculations with a cutoff value of 0.5 to elucidate the nature of interactions governing the hydrogenation of acetone using the CAl_3_MgH_2_^¯^. NCI provides insights into van der Waals interactions, hydrogen bonds, π–π stacking, and other weak interactions, offering a visual representation of non-covalent interactions in a molecular system. The 3D isosurfaces and 2D reduced density gradient (RDG) graphs for all the stationary points along the reaction pathway were generated and are shown in Figure 6, explicitly illustrating the interactions pertinent to bond cleavage and formation. For a comprehensive perspective, the complete 3D isosurfaces and their corresponding RDG graphs are given in Supplementary Figure S4. The atom labels used here follow those in Figure 5. In reactant state A, the presence of blue patches between the acetone and the CAl_3_MgH_2_^¯^ signifies an electrostatic attraction, which initiates the reaction process. As the reaction proceeds in TS1, an increase in blue patches between magnesium and oxygen atoms, as well as between the carbonyl carbon and H1 hydrogen atoms, was observed. These patches indicate a strong electrostatic attraction between the atoms, which facilitates the bond formation process leading to intermediate B. Following the bond formation process in B, no non-covalent interactions are observed, confirming the chemical bond between Mg–O and C–H1. As molecular hydrogen is introduced into C, the presence of green patches between the Mg and H_2_ was observed, indicating van der Waals interactions. This interaction leads to the transition state TS2, where H_2_ is cleaved and added to Magnesium and oxygen. Specifically, the bonding interaction between the O–H3 and between the Mg–H2 enhances the H_2_ heterolytic cleavage, leading to product state D. The presence of blue patches between the Mg and O in product state D indicates a strong attraction between them. Additionally, blue patches between H3 (0.54 |e|) and the central carbon (−2.76 |e|) of CAl_3_MgH_2_^¯^ were observed with a 2.28 Å distance between them, indicating H-bonding. These attractive forces serve as stabilizing factors for product state D, lowering its energy compared to the combined energies of the individual product and CAl_3_MgH_2_^¯^.

To investigate the impact of the CAl_3_MgH_2_^¯^, a comprehensive analysis of the reaction pathway for the hydrogenation of acetone was performed, comparing scenarios both with and without the CAl_3_MgH_2_^¯^. Supplementary Figure S5 illustrates the energy barriers associated with the transition states during this hydrogenation reaction. Additionally, comparison with the zero-point corrected energy profile is given in Supplementary Figure S6 **.** In the absence of a CAl_3_MgH_2_^¯^, the energy barrier for reaching the transition state is relatively high, at 73.82 kcal/mol. In contrast, introducing the CAl_3_MgH_2_^¯^ significantly lowers the energy barriers, revealing a more favorable reaction pathway. Notably, using the CAl_3_MgH_2_^¯^ results in two distinct transition-state energy barriers: the lower of the two, at 6.96 kcal/mol, indicating a markedly reduced activation energy requirement. The second transition state, where the hydrogenation occurs, is at 21.59 kcal/mol. These considerable activation energy barriers demonstrate the effectiveness of the CAl_3_MgH_2_^¯^, which decreases the activation energy barrier for the hydrogenation step by a notable 52.23 kcal/mol compared to the case without a CAl_3_MgH_2_^¯^. Such a substantial drop not only enhances the feasibility of the hydrogenation process but also emphasizes the CAl_3_MgH_2_^¯^ role in facilitating the reaction under more energetically favorable conditions.

## Conclusion

In summary, this study investigates the catalytic properties of the ptC molecule, particularly in acetone hydrogenation. Utilizing computational approaches, the present work systematically examined the catalytic activity of CAl_3_MgH_2_¯ in the hydrogenation of acetone to isopropyl alcohol. The reaction mechanism commences with the carbonyl group of acetone inserted into the magnesium-hydride (Mg–H) bond of the CAl_3_MgH_2_^¯^. Following this step, the reaction progresses through a heterolytic dissociation of the H_2_ molecule into a proton-hydride pair. This key transitional stage is essential, as it drives the hydrogenation reaction and facilitates CAl_3_MgH_2_^¯^ regeneration, ultimately producing the desired isopropyl alcohol. Furthermore, the IRC calculations confirmed that the reaction mechanism follows a minimum-energy pathway, demonstrating strong interconnections between the transition states, reactant, and product. The heterolytic dissociation of H_2_, assisted by the Lewis acid-base pair, is supported by natural atomic charges. The findings underscore the CAl_3_MgH_2_^¯^, demonstrating its ability for acetone hydrogenation, highlighting it as a potential catalyst for hydrogenation reactions. Although several ptC have been observed in the gas phase, effective laboratory synthesis methods are still lacking. Nevertheless, ongoing research in this area is expected to lead to viable synthesis approaches in the future.

## Data Availability

The original contributions presented in the study are included in the article/Supplementary Material, further inquiries can be directed to the corresponding author.
