# Supplementary material for: Catalytic hydrogenation of acetone to isopropyl alcohol with CAl3MgH2 ¯ containing planar tetracoordinate carbon
Source: Front Chem. 2026 Jan 13;13:1750798. doi: 10.3389/fchem.2025.1750798 (PMC12835340; doi:10.3389/fchem.2025.1750798)
Supplement: Supplementary file 1 [file DataSheet1.PDF]

# Catalytic Hydrogenation of Acetone to Isopropyl Alcohol with $CAl_3MgH_2^-$ Containing Planar Tetracoordinate Carbon

Abdul Hamid Malhan[1], Krishnan Thirumoorthy[1,2]*

[1]Department of Chemistry, School of Advanced Sciences, Vellore Institute of Technology, Vellore 632 014, Tamil Nadu, India

[2]School of Computer Science and Engineering, Vellore Institute of Technology, Vellore 632 014, Tamil Nadu, India

***Correspondence:**

Krishnan Thirumoorthy

thirumoorthy.krishnan@vit.ac.in & kthirumoorthy@gmail.com

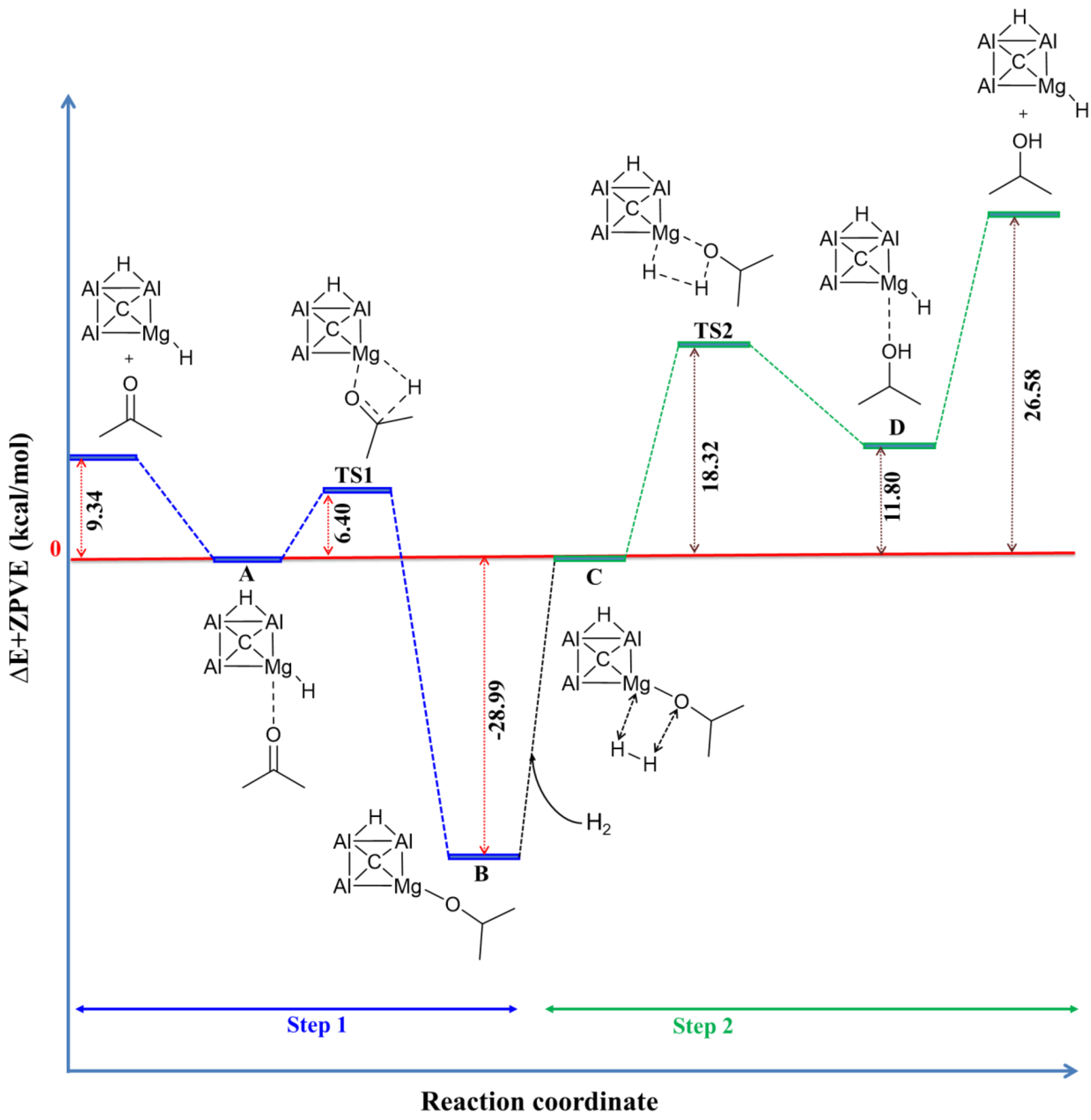


**Figure S1.** Zero-point vibrational corrected total energies profile in kcal/mol for hydrogenation of acetone using $CAl_3MgH_2^-$. The reaction proceeds via two transition states with activation barriers of 6.40 kcal/mol for **TS1** and 18.32 kcal/mol for **TS2**. All energies are calculated at the ωB97XD/def2-TZVPP level.

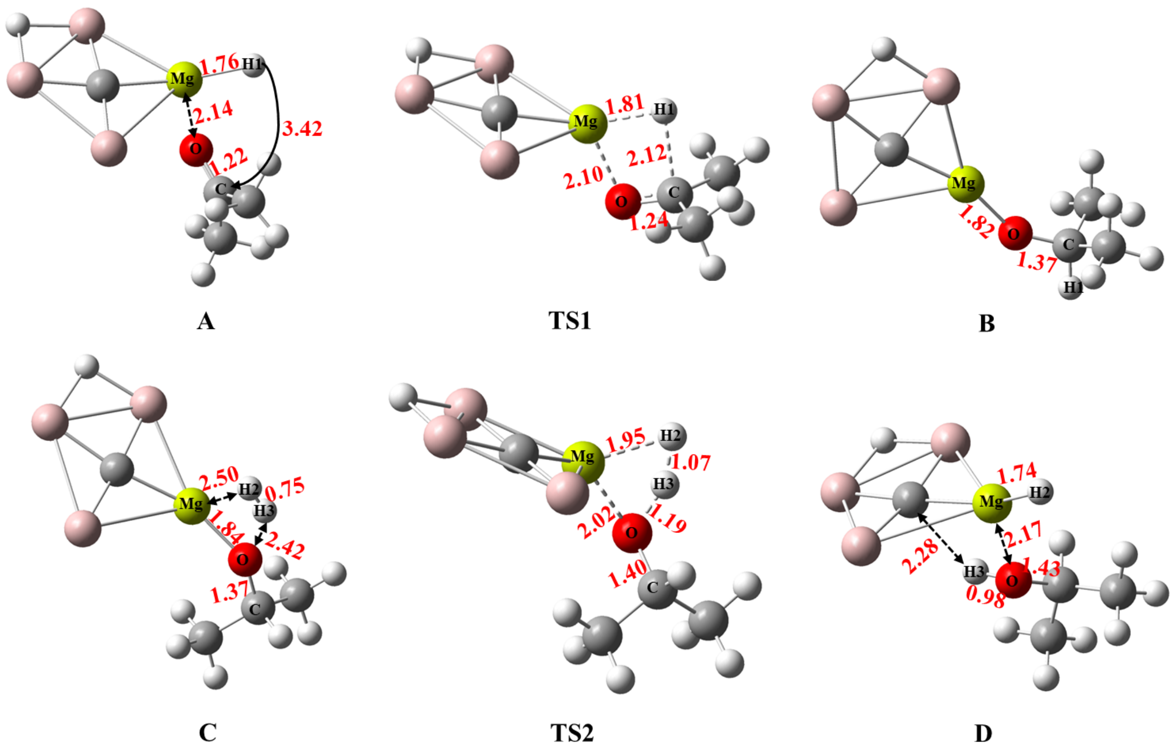


**Figure S2.** Calculated bond lengths in Å for optimized structures of the stationary points involved in the reaction pathway of hydrogenation of acetone using $CAl_3MgH_2^-$ at M06/def2-TZVPP level. The elongation of the Mg–H1 bond in **TS1** confirms the transfer of H1. In **TS2**, the elongation of $H_2$ confirms its cleavage.

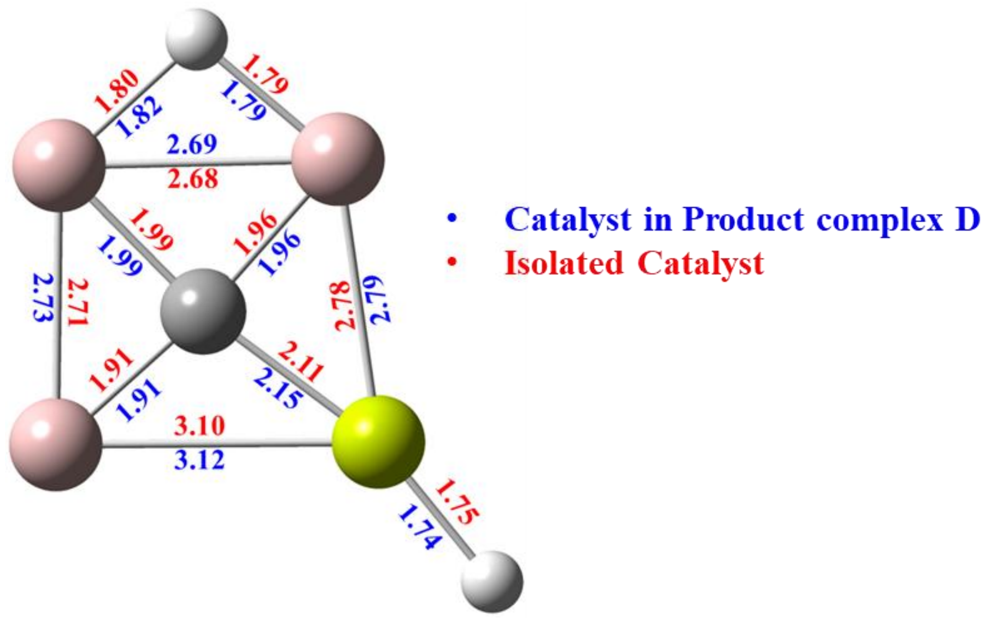


**Figure S3.** Comparison of bond lengths in Å between the isolated $CAl_3MgH_2^-$ and the $CAl_3MgH_2^-$ in product complex **D** at the ωB97XD/def2-TZVPP level.

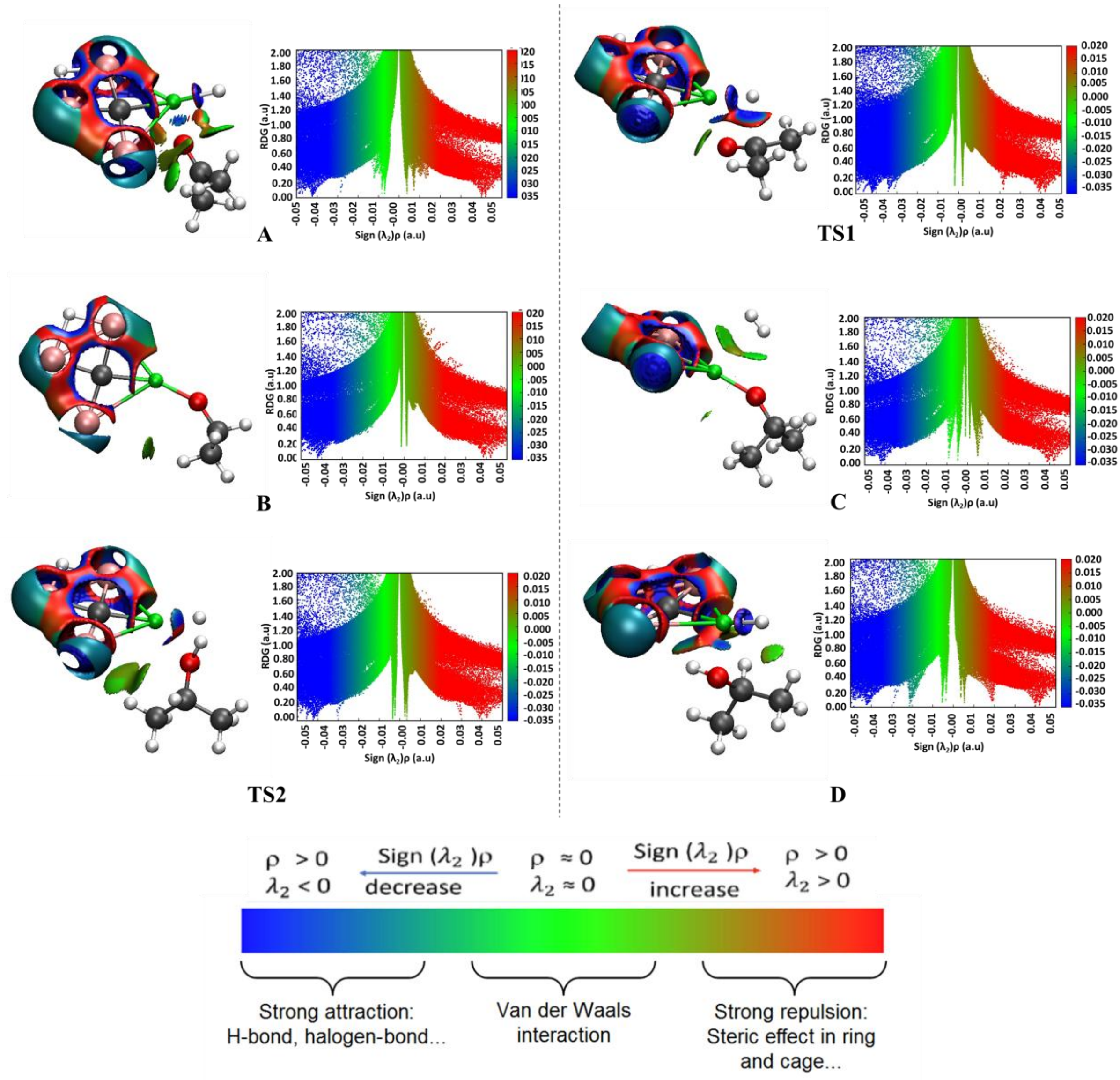


**Figure S4.** Non-covalent interaction, 3D isosurfaces (on left) and 2D reduced density gradient (on right) with complete interaction for optimized geometries of all the stationary points involved in the reaction pathway of hydrogenation of acetone using $CAl_3MgH_2^-$ at ωB97XD/def2-TZVPP level. The reaction initiates with van der Waals interactions (green isosurface) that bring the reactants together in the initial complex. As the system approaches the transition state, these evolve into strong, electrostatic interactions (blue isosurface), which are critical for product formation. Isosurfaces are colored as: strong attraction (blue), van der Waals interaction (green), and repulsive interaction (red).

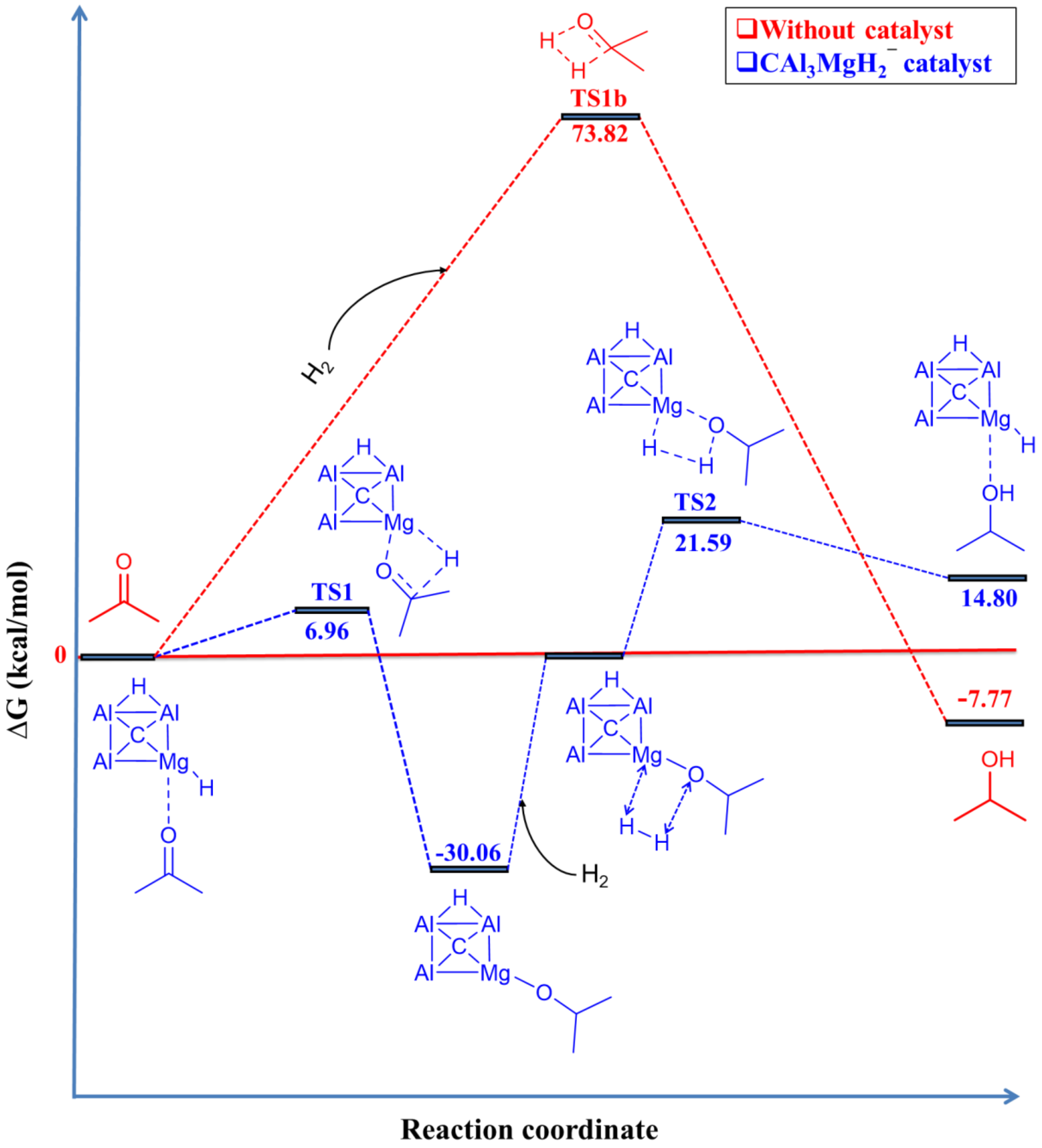


**Figure S5.** Comparison of ΔG in kcal/mol for hydrogenation of acetone with and without $CAl_3MgH_2^-$ at the ωB97XD/def2-TZVPP level.

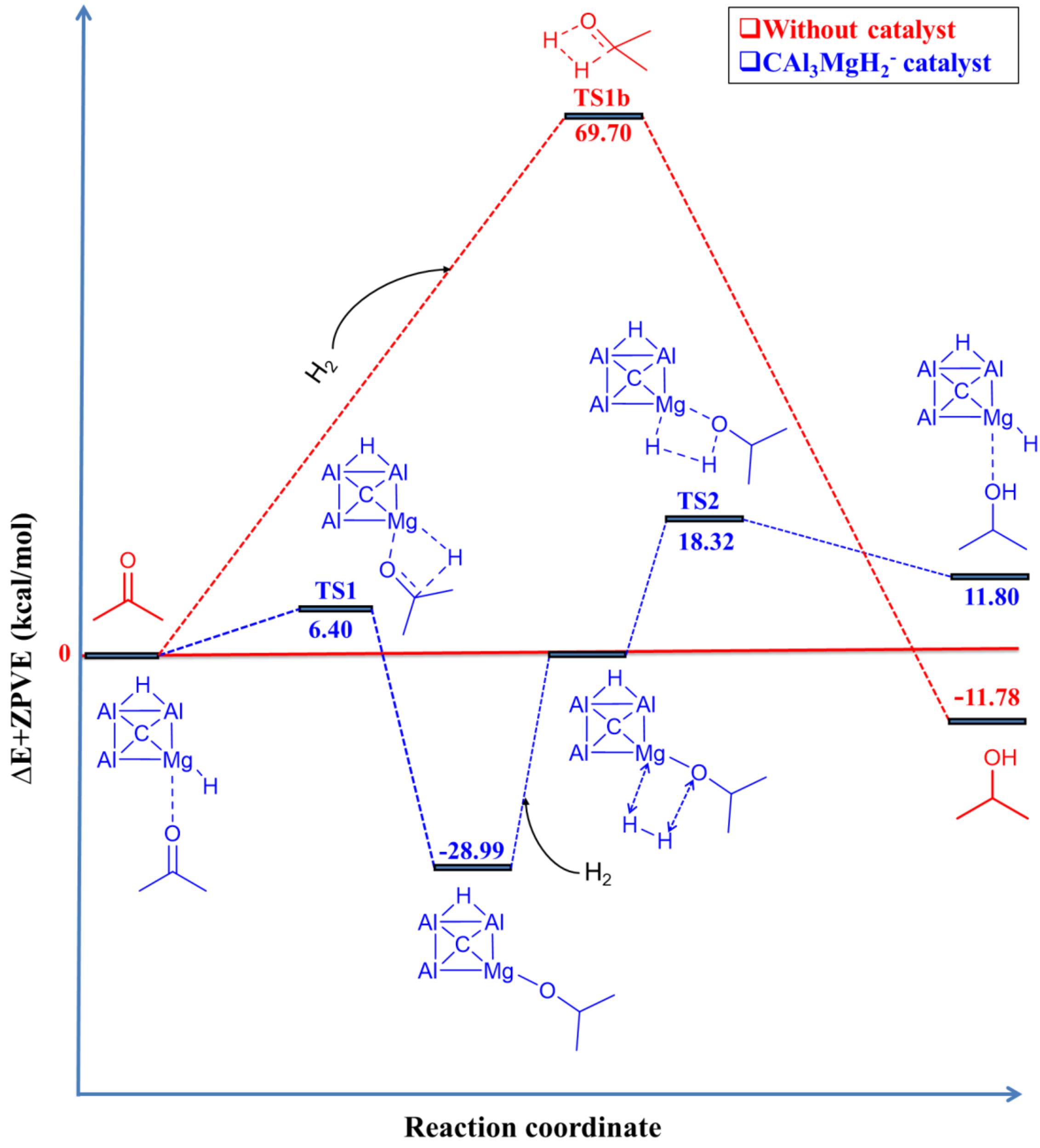


**Figure S6.** Comparison of zero-point vibrational corrected transition state energy barriers in kcal/mol for hydrogenation of acetone with and without $CAl_3MgH_2^-$ at the ωB97XD/def2-TZVPP level.

**Table S1.** Total energy (in a.u)**,** zero-point vibrational energy (ZPVE; in a.u), ZPVE corrected total energy (E+ZPVE; in a.u), relative energy (ΔE + ZPVE; in kcal/mol), and the number of imaginary frequencies (NImag) for optimized structures of the stationary points involved in the reaction pathway of hydrogenation of acetone using $CAl_3MgH_2^-$ at the ωB97XD/def2-TZVPP level.

| | **Energy (a.u)** | **ZPVE (a.u)** | **E+ZPVE (a.u)** | **ΔE+ZPVE (kcal/mol)** | **NImag** |
|---|---|---|---|---|---|
| **A** | -1159.8632 | 0.104872 | -1159.7584 | 0 | 0 |
| **TS1** | -1159.8527 | 0.104507 | -1159.7482 | 6.40 | 1 |
| **B** | -1159.9157 | 0.11118 | -1159.8046 | -28.99 | 0 |
| **C** | -1161.0946 | 0.124026 | -1160.9706 | 0 | 0 |
| **TS2** | -1161.066 | 0.1246 | -1160.9414 | 18.32 | 1 |
| **D** | -1161.0811 | 0.129335 | -1160.9518 | 11.80 | 0 |

**Table S2.** Total energy (in a.u), zero-point vibrational energy (ZPVE; in a.u), ZPVE corrected total energy (E+ZPVE; in a.u), relative energy (ΔE + ZPVE; in kcal/mol), and the number of imaginary frequencies (NImag) for optimized structures of the stationary points involved in the reaction pathway of hydrogenation of acetone using $CAl_3MgH_2^-$ at M06/def2-TZVPP level.

| | **Energy (a.u)** | **ZPVE (a.u)** | **E+ZPVE (a.u)** | **ΔE+ZPVE (kcal/mol)** | **NImag** |
|---|---|---|---|---|---|
| **A** | -1159.7766 | 0.103183 | -1159.6734 | 0 | 0 |
| **TS1** | -1159.769 | 0.103416 | -1159.6656 | 4.89 | 1 |
| **B** | -1159.8257 | 0.10998 | -1159.7157 | -26.54 | 0 |
| **C** | -1161.0005 | 0.122961 | -1160.8776 | 0 | 0 |
| **TS2** | -1160.9707 | 0.123346 | -1160.8474 | 18.95 | 1 |
| **D** | -1160.9848 | 0.127471 | -1160.8573 | 12.73 | 0 |

**Table S3.** Comparison of ΔG and ΔE+ZPVE in kcal/mol obtained at the ωB97XD/def2-TZVPP and M06/def2-TZVPP levels for hydrogenation of acetone using $CAl_3MgH_2^-$.

| Energy (kcal/mol) | | | | |
|---|---|---|---|---|
| | **ωB97XD/def2-TZVPP** | | **M06/def2-TZVPP** | |
| | **ΔG** | **ΔE+ZPVE** | **ΔG** | **ΔE+ZPVE** |
| **A** | 0.00 | 0.00 | 0.00 | 0.00 |
| **TS1** | 6.96 | 6.40 | 5.52 | 4.89 |
| **B** | -30.06 | -28.99 | -27.42 | -26.54 |
| **C** | 0.00 | 0.00 | 0.00 | 0.00 |
| **TS2** | 21.59 | 18.32 | 19.08 | 18.95 |
| **D** | 14.80 | 11.80 | 12.30 | 12.73 |

**Table S4.** Calculated bond lengths in Å of the stationary points involved in the reaction pathway of hydrogenation of acetone using $CAl_3MgH_2^-$ at the ωB97XD/def2-TZVPP and M06/def2-TZVPP levels (Atom labels are followed as given in **Figure S2**).

| | | Mg-H1 | Mg-O | C=O | C-H1 | H2-H3 | Mg-H2 | O-H3 |
|---|---|---|---|---|---|---|---|---|
| **A** | ωB97XD/def2-TZVPP | 1.76 | 2.15 | 1.22 | 3.49 | - | - | - |
| | M06/def2-TZVPP | 1.76 | 2.14 | 1.22 | 3.42 | - | - | - |
| **TS1** | ωB97XD/def2-TZVPP | 1.81 | 2.11 | 1.24 | 2.11 | - | - | - |
| | M06/def2-TZVPP | 1.81 | 2.10 | 1.24 | 2.12 | - | - | - |
| **B** | ωB97XD/def2-TZVPP | - | 1.82 | 1.37 | 1.10 | - | - | - |
| | M06/def2-TZVPP | - | 1.82 | 1.37 | 1.11 | - | - | - |
| **C** | ωB97XD/def2-TZVPP | 2.74 | 1.81 | 1.37 | 1.10 | 0.75 | 2.74 | 2.59 |
| | M06/def2-TZVPP | 2.50 | 1.84 | 1.37 | 1.11 | 0.75 | 2.50 | 2.42 |
| **TS2** | ωB97XD/def2-TZVPP | 1.98 | 2.00 | 1.41 | 1.10 | 1.05 | 1.98 | 1.21 |
| | M06/def2-TZVPP | 1.95 | 2.02 | 1.40 | 1.11 | 1.07 | 1.95 | 1.19 |
| **D** | ωB97XD/def2-TZVPP | 1.74 | 2.17 | 1.43 | 1.10 | - | 1.74 | 0.98 |
| | M06/def2-TZVPP | 1.74 | 2.17 | 1.43 | 1.10 | - | 1.74 | 0.98 |

**Table S5.** Cartesian coordinates of stationary points of hydrogenation of acetone using $CAl_3MgH_2^-$ at the ωB97XD/def2-TZVPP level.

| **A** | | | | **TS1** | | | |
|---|---|---|---|---|---|---|---|
| Mg | 0.306517 | -0.264098 | -1.472858 | Mg | 0.582561 | -0.630860 | -0.427341 |
| C | -1.178441 | 0.084975 | 0.007198 | C | -1.322167 | 0.154754 | -0.006622 |
| Al | -0.450870 | 1.612184 | 0.867679 | Al | -0.971156 | 2.012762 | -0.200355 |
| Al | -1.983661 | -1.573447 | -0.636693 | Al | -1.800350 | -1.730955 | 0.209201 |
| Al | -2.716894 | 0.193163 | 1.245082 | Al | -3.207605 | 0.542592 | 0.447132 |
| H | 1.096487 | 0.279826 | -2.952148 | H | 1.871486 | -0.754066 | -1.694372 |
| H | -3.332690 | -1.352869 | 0.527142 | H | -3.463378 | -1.274887 | 0.609694 |
| C | 3.065530 | -0.274507 | -0.123791 | C | 3.120727 | -0.484378 | -0.008224 |
| C | 4.147321 | -0.906582 | 0.700770 | C | 3.936927 | -1.712325 | -0.304211 |
| H | 3.875361 | -1.921588 | 0.976279 | H | 3.359666 | -2.607635 | -0.091031 |
| H | 5.083666 | -0.903882 | 0.139133 | H | 4.277361 | -1.720511 | -1.336394 |
| H | 4.309801 | -0.304098 | 1.596992 | H | 4.811383 | -1.689811 | 0.355795 |
| O | 2.066575 | -0.907747 | -0.410349 | O | 2.241648 | -0.513514 | 0.865645 |
| C | 3.232278 | 1.149783 | -0.542806 | C | 3.667591 | 0.830900 | -0.491395 |
| H | 4.258460 | 1.497944 | -0.441315 | H | 4.516259 | 1.086153 | 0.153186 |
| H | 2.868084 | 1.268127 | -1.565623 | H | 4.015855 | 0.761140 | -1.518664 |
| H | 2.579495 | 1.757620 | 0.098264 | H | 2.915735 | 1.611416 | -0.402030 |
| **B** | | | | **C** | | | |
| | | | | Mg | 0.369081 | -0.526987 | -0.286793 |
| Mg | 1.614357 | -1.800762 | -1.976206 | C | -1.669125 | -0.179491 | -0.007025 |
| C | -0.426692 | -1.957031 | -2.389971 | Al | -1.777265 | 1.710481 | -0.256526 |
| Al | -1.147918 | -0.558022 | -1.310891 | Al | -1.720527 | -2.132360 | 0.194781 |
| Al | 0.193197 | -3.423791 | -3.540418 | Al | -3.611868 | -0.238027 | 0.394000 |
| Al | -2.245242 | -2.424063 | -3.033733 | H | 3.863963 | 0.798575 | -0.915219 |
| H | 4.887605 | -0.503838 | -0.428246 | H | -3.451058 | -2.064319 | 0.547098 |
| H | -1.460677 | -3.756278 | -4.048288 | C | 3.347370 | 0.315328 | -0.067031 |
| C | 3.803214 | -0.296958 | -0.404084 | C | 4.265347 | -0.794238 | 0.447036 |
| C | 3.340751 | -0.464861 | 1.043707 | H | 4.406063 | -1.548826 | -0.327639 |
| H | 3.489726 | -1.496929 | 1.364319 | H | 5.244310 | -0.406088 | 0.741557 |
| H | 3.886666 | 0.194356 | 1.724208 | H | 3.808057 | -1.279951 | 1.312932 |
| H | 2.274617 | -0.233160 | 1.119824 | O | 2.149117 | -0.202182 | -0.495020 |
| O | 3.153704 | -1.176901 | -1.236955 | C | 3.141382 | 1.386994 | 1.003916 |
| C | 3.605806 | 1.147262 | -0.866033 | H | 2.666843 | 0.943247 | 1.884016 |
| H | 2.544237 | 1.406793 | -0.822843 | H | 4.086334 | 1.840711 | 1.314884 |
| H | 4.159460 | 1.853539 | -0.241285 | H | 2.485937 | 2.172772 | 0.625301 |
| H | 3.942460 | 1.257029 | -1.897935 | H | 1.031518 | -0.413143 | -2.817568 |
| | | | | H | 0.320584 | -0.516374 | -3.027710 |
| **TS2** | | | | **D** | | | |

| | | | | | | | |
|---|---|---|---|---|---|---|---|
| Mg | 0.145549 | -0.988601 | -0.587191 | Mg | -1.295107 | 1.400275 | -1.143303 |
| C | -1.591552 | 0.106122 | -0.170339 | C | -1.349725 | 0.329296 | 0.722061 |
| Al | -1.085078 | 1.844850 | -0.756791 | Al | -0.325309 | 1.514675 | 1.819500 |
| Al | -2.228604 | -1.641652 | 0.441305 | Al | -2.641779 | -0.850176 | -0.162448 |
| Al | -3.369202 | 0.786026 | 0.376859 | Al | -1.466530 | -0.921543 | 2.260521 |
| H | 2.623887 | 0.991035 | -0.786333 | H | -0.416870 | -1.389628 | -2.589661 |
| H | -3.779424 | -0.944499 | 0.899203 | H | -2.659021 | -1.890625 | 1.291653 |
| C | 2.877854 | 0.266041 | 0.001914 | C | 0.504126 | -0.797109 | -2.638697 |
| C | 4.356428 | -0.075799 | -0.115580 | C | 1.700119 | -1.703147 | -2.411353 |
| H | 4.566572 | -0.527383 | -1.085733 | H | 1.629909 | -2.198102 | -1.441684 |
| H | 4.975039 | 0.817656 | -0.007862 | H | 1.745491 | -2.473320 | -3.183012 |
| H | 4.634857 | -0.790647 | 0.661466 | H | 2.624587 | -1.123881 | -2.437895 |
| O | 2.103619 | -0.899704 | -0.174229 | O | 0.445743 | 0.198323 | -1.611869 |
| C | 2.542451 | 0.894321 | 1.344515 | C | 0.556239 | -0.064860 | -3.962742 |
| H | 2.770260 | 0.194067 | 2.150860 | H | 1.458907 | 0.545648 | -4.022364 |
| H | 3.116524 | 1.809541 | 1.500879 | H | 0.559681 | -0.778326 | -4.787521 |
| H | 1.481856 | 1.145451 | 1.395760 | H | -0.307227 | 0.591568 | -4.073304 |
| H | 1.901532 | -1.221098 | -1.322611 | H | 0.304471 | -0.221725 | -0.741163 |
| H | 1.279504 | -1.486669 | -2.128189 | H | -1.547910 | 2.722628 | -2.246389 |

**Table S6.** Cartesian coordinates of stationary points of hydrogenation of acetone using $CAl_3MgH_2^-$ at M06/def2-TZVPP level.

| **A** | | | | **TS1** | | | |
|---|---|---|---|---|---|---|---|
| Mg | -0.111482 | -0.993434 | 0.999470 | Mg | 0.381042 | -0.365542 | -0.428515 |
| C | 1.515702 | 0.101747 | 0.218727 | C | -1.631601 | 0.082929 | -0.101480 |
| Al | 0.823771 | 1.862221 | 0.418656 | Al | -1.517875 | 1.985686 | -0.107373 |
| Al | 2.282738 | -1.645961 | -0.209970 | Al | -1.833203 | -1.872224 | -0.091750 |
| Al | 3.166039 | 0.881374 | -0.534262 | Al | -3.574246 | 0.162915 | 0.226145 |
| H | -1.082233 | -1.387448 | 2.416457 | H | 1.762021 | -0.190294 | -1.595563 |
| H | 3.743218 | -0.821253 | -0.858869 | H | -3.572657 | -1.690173 | 0.207933 |
| C | -2.727851 | -0.107896 | -0.300543 | C | 2.848551 | 0.027306 | 0.214062 |
| C | -3.769313 | -0.153124 | -1.369594 | C | 3.779507 | -1.097917 | -0.113324 |
| H | -3.485655 | -0.846560 | -2.157098 | H | 3.259647 | -2.050991 | -0.040568 |
| H | -4.729207 | -0.444565 | -0.937730 | H | 4.212266 | -0.982670 | -1.104916 |
| H | -3.904616 | 0.848746 | -1.783858 | H | 4.590139 | -1.084829 | 0.624815 |
| O | -1.728042 | -0.797205 | -0.384883 | O | 1.904494 | -0.147169 | 0.996452 |
| C | -2.930615 | 0.821907 | 0.839663 | C | 3.320748 | 1.408199 | -0.115670 |
| H | -3.927205 | 1.259803 | 0.863959 | H | 4.098692 | 1.674325 | 0.610049 |
| H | -2.702104 | 0.293814 | 1.771037 | H | 3.750239 | 1.454518 | -1.114297 |
| H | -2.178127 | 1.623687 | 0.757168 | H | 2.504793 | 2.122901 | -0.025998 |
| **B** | | | | **C** | | | |
| | | | | Mg | -0.288576 | -0.317219 | 0.548671 |
| Mg | -0.308431 | 0.165161 | -0.000122 | C | 1.682353 | 0.081747 | 0.043180 |
| C | 1.760056 | 0.103653 | -0.000029 | Al | 1.705696 | 1.986148 | 0.227373 |
| Al | 2.177646 | 1.973153 | -0.000040 | Al | 1.796273 | -1.883543 | -0.064791 |
| Al | 1.447371 | -1.848987 | 0.000055 | Al | 3.563219 | 0.091620 | -0.574178 |
| Al | 3.691548 | -0.345404 | 0.000072 | H | -4.026213 | 0.567459 | 0.776725 |
| H | -3.966416 | 1.371264 | -0.000058 | H | 3.471255 | -1.758371 | -0.620225 |
| H | 3.193408 | -2.126472 | -0.000016 | C | -3.231425 | 0.190412 | 0.102708 |
| C | -3.347785 | 0.451699 | 0.000007 | C | -3.740813 | -1.103483 | -0.516085 |
| C | -3.713981 | -0.339107 | 1.247681 | H | -3.890479 | -1.857896 | 0.257817 |
| H | -3.451942 | 0.227276 | 2.142918 | H | -4.681226 | -0.960596 | -1.055786 |
| H | -4.780387 | -0.580045 | 1.285949 | H | -2.994630 | -1.489777 | -1.218991 |
| H | -3.147705 | -1.276795 | 1.262870 | O | -2.082760 | -0.026751 | 0.818631 |
| O | -2.020941 | 0.786317 | -0.000226 | C | -3.018598 | 1.242393 | -0.975808 |
| C | -3.714307 | -0.339509 | -1.247316 | H | -2.273119 | 0.884246 | -1.695899 |
| H | -3.148026 | -1.277196 | -1.262354 | H | -3.938104 | 1.469014 | -1.523092 |
| H | -4.780722 | -0.580466 | -1.285222 | H | -2.637021 | 2.164855 | -0.533625 |
| H | -3.452509 | 0.226589 | -2.142803 | H | -0.820797 | 0.049789 | 2.883009 |
| | | | | H | -0.084389 | -0.016668 | 3.026380 |
| **TS2** | | | | **D** | | | |

| | | | | | | | |
|---|---|---|---|---|---|---|---|
| Mg | 0.113039 | -0.997329 | -0.619448 | Mg | 0.063746 | -1.387490 | 0.952591 |
| C | -1.627196 | 0.062224 | -0.200191 | C | -1.480270 | -0.187072 | 0.105411 |
| Al | -1.122360 | 1.794997 | -0.824221 | Al | -2.104285 | -1.425451 | -1.221648 |
| Al | -2.248917 | -1.698733 | 0.414627 | Al | -1.087684 | 1.107277 | 1.529369 |
| Al | -3.401184 | 0.738444 | 0.345674 | Al | -2.829190 | 1.114235 | -0.543370 |
| H | 2.499603 | 1.021374 | -0.740163 | H | 2.140714 | 0.989059 | 0.811336 |
| H | -3.804558 | -0.999838 | 0.874050 | H | -2.345579 | 2.192143 | 0.843209 |
| C | 2.797431 | 0.294981 | 0.038131 | C | 2.469921 | 0.417171 | -0.069662 |
| C | 4.279541 | 0.018773 | -0.101558 | C | 2.797009 | 1.373442 | -1.189898 |
| H | 4.500418 | -0.411289 | -1.079752 | H | 1.929944 | 1.990151 | -1.436623 |
| H | 4.866438 | 0.932579 | 0.015625 | H | 3.614594 | 2.037466 | -0.903628 |
| H | 4.592107 | -0.697554 | 0.662488 | H | 3.092274 | 0.819210 | -2.083731 |
| O | 2.074585 | -0.899268 | -0.137890 | O | 1.403293 | -0.446489 | -0.468108 |
| C | 2.461114 | 0.883110 | 1.389527 | C | 3.627379 | -0.469393 | 0.308051 |
| H | 2.736773 | 0.176687 | 2.176702 | H | 3.954485 | -1.047604 | -0.559178 |
| H | 2.996978 | 1.819847 | 1.556408 | H | 4.466272 | 0.129154 | 0.665968 |
| H | 1.390354 | 1.083551 | 1.470119 | H | 3.336553 | -1.168921 | 1.093445 |
| H | 1.902806 | -1.183762 | -1.278630 | H | 0.619652 | 0.079118 | -0.734233 |
| H | 1.281998 | -1.414761 | -2.123150 | H | 0.795773 | -2.732909 | 1.770452 |

**Table S7.** Cartesian coordinates of stationary points of hydrogenation of acetone without $CAl_3MgH_2^-$ at ωB97XD/def2-TZVPP level.

| **A-2** | | | | **TS1b** | | | |
|---|---|---|---|---|---|---|---|
| C | 0.114720 | 0.113210 | -0.000008 | C | 0.000001 | 0.115491 | 0.142023 |
| C | 1.619702 | 0.235370 | -0.000116 | C | -1.278202 | -0.663154 | -0.069626 |
| H | 1.911234 | 1.281988 | -0.000254 | H | -2.134807 | -0.072661 | 0.243691 |
| H | 2.034633 | -0.264657 | 0.877157 | H | -1.353713 | -0.825593 | -1.147357 |
| H | 2.034548 | -0.264884 | -0.877296 | H | -1.271231 | -1.628304 | 0.435680 |
| O | -0.600036 | 1.083825 | 0.000132 | O | 0.000021 | 1.392149 | -0.222599 |
| H | -3.916733 | 0.494720 | -0.000422 | H | -0.000005 | 0.166720 | 1.595663 |
| H | -3.205383 | 0.717672 | -0.000290 | H | 0.000005 | 1.014381 | 1.104464 |
| C | -0.442958 | -1.289746 | 0.000021 | C | 1.278184 | -0.663184 | -0.069623 |
| H | -1.529113 | -1.265194 | 0.000303 | H | 1.353749 | -0.825492 | -1.147370 |
| H | -0.085483 | -1.832427 | -0.877222 | H | 2.134799 | -0.072766 | 0.243811 |
| H | -0.085015 | -1.832615 | 0.876956 | H | 1.271138 | -1.628397 | 0.435563 |
| **B-2** | | | | | | | |
| C | 0.012518 | 0.141335 | -0.355186 | | | | |
| C | -1.242926 | -0.645167 | -0.037330 | | | | |
| H | -2.128524 | -0.035827 | -0.212699 | | | | |
| H | -1.304380 | -1.537308 | -0.660785 | | | | |
| H | -1.238213 | -0.951583 | 1.009755 | | | | |
| O | -0.021298 | 1.307362 | 0.456579 | | | | |
| C | 1.277071 | -0.666403 | -0.106043 | | | | |
| H | 1.325676 | -0.976647 | 0.938790 | | | | |
| H | 1.299021 | -1.557427 | -0.735070 | | | | |
| H | 2.169315 | -0.078305 | -0.331600 | | | | |
| H | 0.772635 | 1.816188 | 0.297215 | | | | |
| H | -0.016345 | 0.435520 | -1.412419 | | | | |

**Table S8.** Cartesian coordinates of the $CAl_3MgH_2^-$ at the ωB97XD/def2-TZVPP level.

| **$CAl_3MgH_2^-$** | | | |
|---|---|---|---|
| Mg | 2.096702 | 0.097107 | 0.000000 |
| C | 0.002173 | 0.128704 | 0.000000 |
| Al | -0.333218 | 2.007746 | 0.000000 |
| Al | 0.205970 | -1.821286 | 0.000000 |
| Al | -1.951355 | -0.218565 | 0.000000 |
| H | 3.752414 | 0.609409 | 0.000000 |
| H | -1.549589 | -2.016967 | 0.000000 |
